# Integrating Molecular Biology and Bioinformatics Education

**DOI:** 10.1515/jib-2019-0005

**Published:** 2019-05-30

**Authors:** Boas Pucker, Hanna Marie Schilbert, Sina Franziska Schumacher

**Affiliations:** Genetics and Genomics of Plants, CeBiTec and Faculty of Biology, Bielefeld University, Bielefeld, Germany; Bielefeld University, Bielefeld, Germany

**Keywords:** bioinformatics, genome assembly, genome research, molecular biology, NGS, RNA-Seq, sequencing technologies, teaching, transcriptome assembly, variant calling

## Abstract

Combined awareness about the power and limitations of bioinformatics and molecular biology enables advanced research based on high-throughput data. Despite an increasing demand of scientists with a combined background in both fields, the education of dry and wet lab subjects are often still separated. This work describes an example of integrated education with a focus on genomics and transcriptomics. Participants learned computational and molecular biology methods in the same practical course. Peer-review was applied as a teaching method to foster cooperative learning of students with heterogeneous backgrounds. The positive evaluation results indicate that this approach was accepted by the participants and would likely be suitable for wider scale application.

## 1 Introduction

There is an increasing demand from academia and industry for life scientists with a strong combined background in both, molecular biology and bioinformatics [1], [2], [3]. Although there are numerous study programs which are addressing this demand for bioinformaticians [3], [4], single courses at a university are usually focused either on the wet lab or the dry lab independently. Frequently, lecturers with a bioinformatics background teach the bioinformatics aspect, while biologists teach the molecular biology part. Probably as a result of this strict separation, many students tend to be substantially more interested in one aspect of their program than the other. Focusing on bioinformatics can cause a lack of knowledge about biology and vice versa. Truly combining both aspects in a single course by looking at both sides of an experiment could help to reduce the separation of wet lab and dry lab thinking, finally leading to a new awareness [5]. In addition, bioinformatics students as well as life science students could be interested in such a course thus facilitating exchange and cooperative learning between students with different educational backgrounds [6].

Combining substantial knowledge and experience about bioinformatics and biology in a single person would lead to the training of highly skilled and urgently needed scientists [1], [3], [7], [8]. These scientists are not just able to communicate efficiently with scientists from both fields, but are even able to address most challenges found in both the wet and dry lab components of a project [9]. The awareness of possibilities and limitations of methods in both fields is very important for successful projects. Due to a continuous increase in publicly available data sets, the ability to harness the power of computational tools effectively is gaining relevance [9]. The potential utility of a scientist trained in both wet and dry lab subjects, along with improvements in public access to data, highlights the need for research into determining the best approach for providing such a combined educational program. As the range of different topics that could be included in a bioinformatics education program is particularly broad [10] it is necessary to focus upon a certain subject area when investigating best practices.

This work describes the concept and content of two courses, which are committed to integrate molecular biology and bioinformatics education with a specific focus on genomics and transcriptomics. The results presented here are the experiences of individuals involved in designing, running, and taking these courses over the last two years. Our intention is to provide an inspiring and practical example of an approach which could be utilized by lecturers at the university level.

## 2 Results

### 2.1 Concept of Complementary Courses

**Figure 1:**
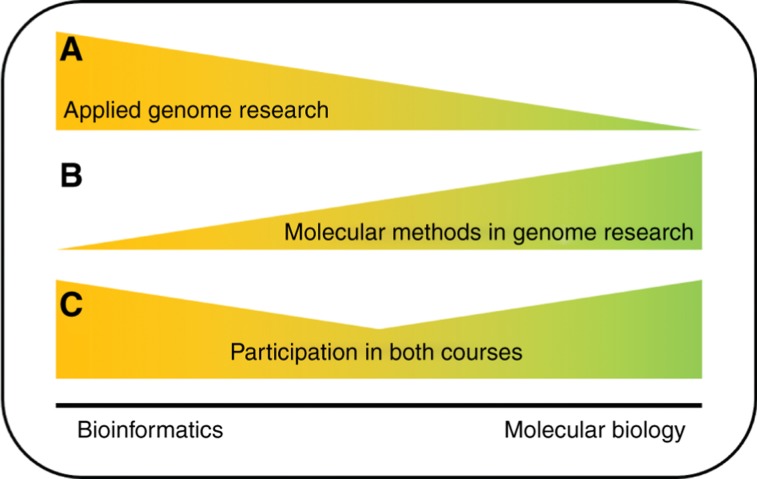
Course content focus. The complementing design of two courses integrates bioinformatics and molecular biology education. The proportion of bioinformatics content (yellow) and molecular biology content (green) is illustrated for the courses “Applied Genome Research” (A), “Molecular Methods in Genome Research” (B), and for the combination of both courses (C).

This approach to educate students about the wet lab and dry lab aspects of genome research was developed over the last three years and resulted in two courses which complement each other. Firstly, a course about bioinformatics methods (“Applied Genome Research”, https://github.com/bpucker/AppliedGenomeResearch) was substantially enriched with molecular biology content. Secondly, a molecular biology course was enriched with bioinformatics methods to mirror this concept from the wet lab side (“Molecular Methods in Genome Research”, https://github.com/bpucker/MolecularMethodsInGenomeResearch) (Figure 1). Both courses were designed to attract both bioinformatics and life science students in order to increase their engagement with the other field. Further reinforcing the combined approach, exercises in these courses often require knowledge from both fields.

### 2.2 Course 1: Applied Genome Research

**Figure 2:**
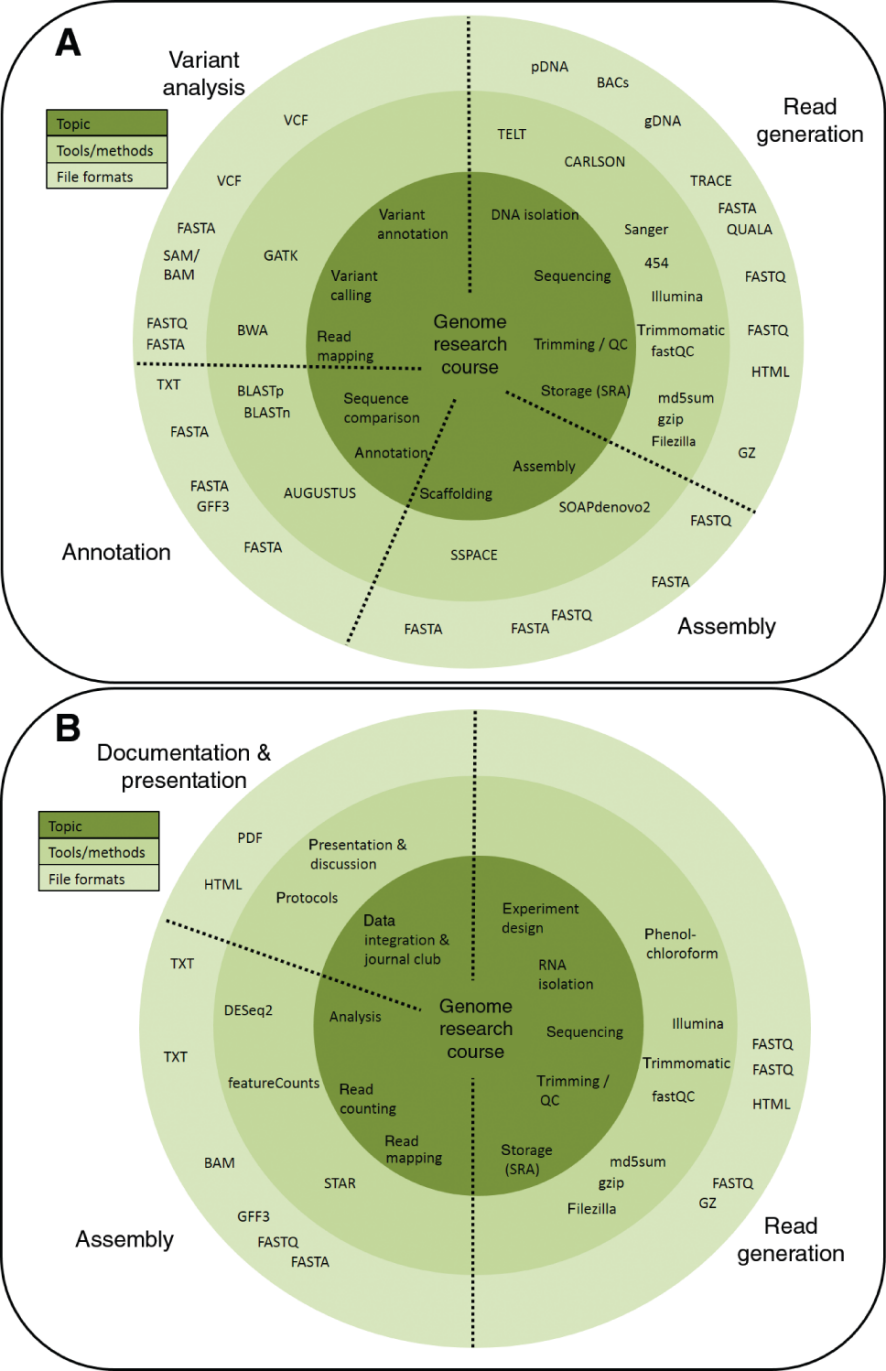
Applied Genome Research course content overview. The content of this course is distributed over two weeks: one genomic (A) and one transcriptomic (B) week. The inner circle contains topics, the middle circle contains methods and tools, and the outer circle contains materials and file formats. Abbreviations in these figures (excluding tool and file format names): plasmid DNA (pDNA), bacterial artificial chromosome (BAC), genomic DNA (gDNA), Sequence Read Archive (SRA).

The content of this course is separated into a genomics section and a transcriptomics section (Figure 2). There are also three layers involved in this teaching process: general concept/aim, method/tool, and the material/data type. Since some participants have a pure life science background without any prior knowledge in bioinformatics, a short introduction into Linux was given to achieve familiarity with using a command line interface.

Starting the genomics section with the biological challenge of isolating DNA (plasmids, BACs, genomic DNA) of sufficient quality and quantity, the introduction provides background knowledge about sequencing technologies and relevant file formats. The next steps were composed to reconstitute a real workflow in plant genome research [11] including preparation for submission to a standard repository like the Sequence Read Archive [12], trimming of reads via trimmomatic [13], and quality control via fastQC [14].

Since the computation of a plant genome assembly consumes a substantial amount of time and computational resources, the read data set was reduced to a subset just representing about 3 Mbp of the *Arabidopsis thaliana* Niederzenz-1 (Nd-1) genome sequence [11]. Generating an assembly via SOAPdenovo2 [15] and assessing different ways of scaffolding were the next steps. Exercises and discussions about the performance of different tools and the impact of certain parameters were a central teaching focus. AUGUSTUS [16] was applied for structural gene prediction and BLAST [17] was used in supplied Python scripts [11] to transfer functional annotations to the predicted genes. This whole process of genome annotation was accompanied by discussions about the biological interpretation of results, possible pitfalls, alternatives, and next steps.

As high quality reference genome sequences become available, *de novo* assemblies are often replaced by read mappings against an existing reference thus enabling the investigation of populations [18]. Therefore, the next step was the mapping of the above-described Nd-1 reads via BWA MEM [19] against a reference sequence (TAIR10, [20]). Variants were called via GATK [21] and functional implications were predicted using SnpEff [22] and NAVIP (https://doi.org/10.1101/596718). The tools applied in this course are not necessarily the best performing ones for a specific step, but overall provide the experience of running a complete genomics workflow. While initially the usage of tools is explained in detail, students were continuously trained to retrieve usage information from the documentation of these tools to facilitate independent application of various bioinformatic tools.

The transcriptomics part started with an introduction to experiment design and RNA isolation. Differences between DNA and RNA processing were discussed. Redundant steps between the genomics and transcriptomics parts were included to reinforce learning through repetitions. The mapping of RNA-Seq reads via STAR [23] and the quantification of gene expression with featureCounts [24] were the first practical steps. To reduce the computational costs associated with the RNA-Seq read mapping, replicates of the resulting count tables were randomly generated using a customized Python script. Afterwards, DESeq2 [25] was applied for statistical analysis of the observed expression values. Different ways to interpret the results were discussed and participants engaged with databases of different model organisms including Araport11 and TAIR10. Besides gene expression analysis, RNA-Seq reads were also used for a transcriptome assembly workflow [26]. Differences between genome and transcriptome assemblies were discussed to identify unique challenges.

Finally, participants demonstrated their enhanced understanding of genomics and transcriptomics in a journal club during the discussion of scientific publications. Each participant gave an approximately 15 minute talk about a recent publication in the field to complete this course. In addition, participants had to write a report about the course topics, applied methods, and results (S1 Text). The report quality was increased by double blind peer-review thus each participant assessed and commented on two reports [27]. This assessment of reports facilitated a stronger engagement with the content thus leading to a deeper understanding. Additionally, important skills were improved e.g. providing constructive criticism about a scientific work.

### 2.3 Course 2: Molecular Methods in Genome Research

**Figure 3:**
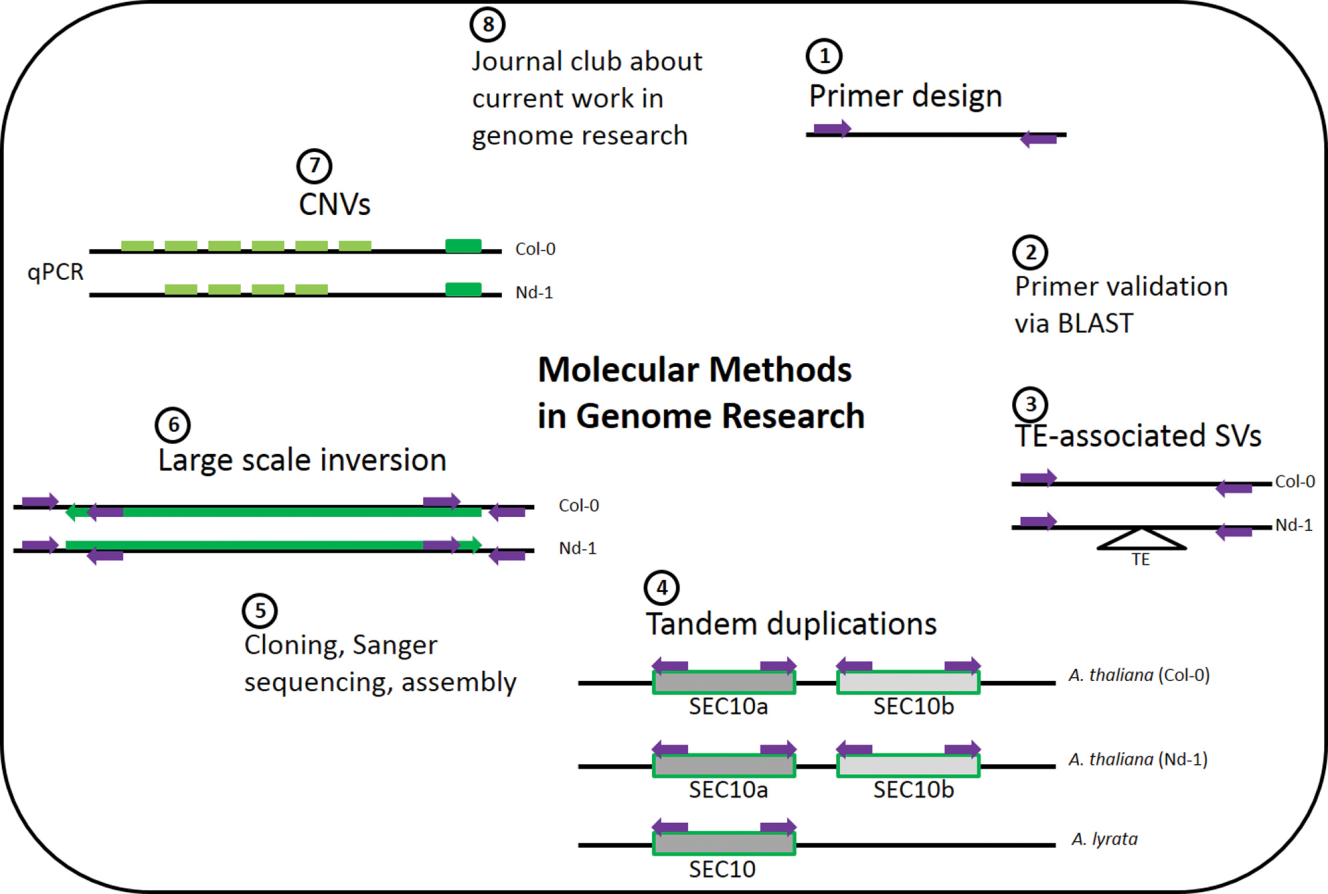
Molecular Methods in Genome Research course content overview. Course content overview displays the interleaved use of bioinformatics and molecular biology.

This course was about validating bioinformatics findings through wet lab experiments (Figure 3). Structural variations between *A. thaliana* accessions were previously identified [11] and provided as a starting point. Participating students had a background in biology or bioinformatics without prior knowledge about the other field. Students selected appropriate targets and subjected them to bioinformatic tools and approaches to prepare their experiments. For example, participants extracted the sequence of target regions from assemblies, designed oligonucleotides for PCR assays, and validated these oligonucleotide combinations via customized Python scripts based on sequence alignments. These initial steps enabled the acquisition of basic Linux skills. Participants became familiar with running scripts on the command line. As all participants worked on different loci, the following molecular biology experiments were unique as well. Moreover, all participants were working on a unique set of *A. thaliana* accessions taken from the Nordborg collection [28]. As a result, all participants were generating new scientific knowledge contributing to the field of *Arabidopsis* genomics. To bridge the time for ordered oligonucleotides to arrive, some experiments derived from recent genome research projects [11], [29], [30], [31] were repeated on different biological material. Therefore, participants were carrying out actual research with unknown outcome. At the same time, it was possible to include positive controls.

The results were documented online in a wiki (S2 Text) to facilitate cooperative learning by avoiding isolated lab reports. Students were able to directly interact with each others’ work by commenting on the wiki pages. Basic knowledge about HTML and wiki code was provided during seminars. Peer-review was applied to enhance the quality of individual wiki pages thus each participant was assessing the wiki pages of two other students. The use of a wiki requires some work during setup, but enables the compliance with data protection laws, which might differ between universities and countries.

### 2.4 Lessons Learned – Evaluation Results

Participants were asked to provide feedback about these courses. Some evaluation results of “Applied Genome Research” were previously described and discussed [27]. Small course sizes (n < 10) prevented detailed statistical analyses of these results, but response rates of usually over 50% and repetitions of the courses allowed inference of general trends. All participants would recommend these courses to their fellow students. Usage of peer-review to improve the quality of reports or wiki pages, respectively, was seen as a good approach, but the reviewer qualification was reported as a main concern. Nevertheless, participants stated that they improved several skills like critical reading and providing feedback through this process. In addition, this repetition of the course content was appreciated.

## 3 Discussion

The presented courses provide an example for interdisciplinary and innovative teaching methods. Their evaluation indicated participants’ satisfaction and a good match with participants’ expectations. More detailed evaluation results of two iterations of the “Applied Genome Research” course were described before with focus on peer-review as a teaching method [27]. In combination with novel insights of more recent iterations, a more controlled version of this process could further increase the benefit. Currently, a strong heterogeneity in the review quality is a major concern brought up by several participants. Implementing a system in which all reports are evaluated by many peers as it is postulated by many open science movements (reviewed in [32]), could be a solution. Reviewers might be more motivated thus producing better reports when they know that their reports will be published. In addition, errors in reviews could be identified and removed if a large number of peers are inspecting them.

Another important point revealed by the evaluation is the proximity to actual research. Students appeared to be more motivated when working on their own experiments and this has been reported before by others [33]. Despite learning valuable skills about experiment design and project management, an extended independence during practical courses could increase the overall interest of students in a subject as well as their self-confidence. However, this comes with higher costs of these innovative experiments, financially and in it becoming more time consuming to prepare for. One example is the need for custom oligonucleotides per student as described for the “Molecular Methods in Genome Research” course. To enable similar courses without external funding, the accumulation of material over years could be the way to go. Some of the materials e.g. oligonucleotides could be used again for following repetitions of a course. Students within one cohort could perform individual experiments, while these experiments are derived from a pool of experiments repeated in every year. In addition, it is feasible that experiments are repeated within one course thus having randomly selected students unknowingly perform the same experiments. This approach enables the validation of results through replicates and can save resources. As all responding students are recommending this course, it is highly likely that the course will be successful when repeated.

Students appreciated the integration of innovative teaching methods. The majority liked the replacement of classical lab reports by digital documentation in a wiki. Although, the application of a wiki as a teaching method is not completely novel [34], it is rarely used in practical courses. It makes students think about displaying their results in an engaging way and connecting them to existing knowledge via hyperlinks. Learning some HTML basics during the wiki construction is an additional benefit, because students learn the concept of markup languages and the foundation for the development of websites. Finally, the interaction between students with different backgrounds during the peer-review process enables additional exchange and cooperative learning. This provides an opportunity for students to practice science communication very early during their education. They can develop skills that are beneficial and required for future projects when working in a team.

Although, this example is focused on the combination of bioinformatics with molecular biology, there are other fields in the life sciences, which would benefit from computational methods as well. Therefore, this description is intended to inspire the development of similar courses in other life science fields to facilitate integrated teaching. Updates of the presented courses will be described on the respective github pages:

https://github.com/bpucker/APPLS,

https://github.com/bpucker/AppliedGenomeResearch, and https://github.com/bpucker/MolecularMethodsInGenomeResearch.
