# Supplementary material for: Integrating Molecular Biology and Bioinformatics Education
Source: J Integr Bioinform. 2019 May 30;16(3):20190005. doi: 10.1515/jib-2019-0005 (PMC6798849; doi:10.1515/jib-2019-0005)
Supplement: Supplementary file 1 [file jib-16-20190005-s001.docx]

**Supporting information captions**

**S1 Text: Report about ‘Applied Genome Research’ by Hanna Marie Schilbert.** This example provides a more detailed impression of the course content and also illustrates how participants perceive it.

**S2 Text: Report about ‘Molecular Methods in Genome Research’ by Sina Franziska Schumacher.** This example provides a more detailed impression of the course content and also illustrates how participants perceive it.
